# The Effects of Different Remedies in Diabetes*A Case of Sacharine Diabetes, with the observations on the Results of Treatment. By Charles Frick, M.D., Baltimore. American Jour. of Medical Sciences, July, 1851.

**Published:** 1853-08

**Authors:** 


Half-yearly Abstract of Med. Sciences.

*The Effects of Different Remedies in Diabetes.**

The subject of Dr. Frick's observations was placed under peculiarly favorable circumstances for ensuring all necessary certainty. He was a prisoner in the Maryland Penitentiary, in the prime of life, not fleshy, but healthy and strong in appearance. The case itself presented no unusual peculiarities.

In the course of the investigation, abundant proof of the general and not local character of the disease was obtained, sugar being found not only in the urine but also in the sweat, fæces, chyme, bronchial mucus, saliva, as well as in the blood and in the purulent matter of an abscess in the hand. Sugar was also found in the ordinary excretions, and in considerable, though in diminished quantities, when all sugar or sacharifiable substances were carefully excluded from the food; it was even found in the chyme when the previous meal had consisted of meat and eggs alone.

Dr. Frick's conclusions upon the influence of diet are somewhat surprising. In the matter of *drinks* he finds it not necessary to subject the patient to any privations. He found, indeed, that the *specific gravity* of the urine varied according to the amount of

* A Case of Sacharine Diabetes, with the observations on the Results of Treatment. By Charles Frick, M.D., Baltimore. American Jour. of Medical Sciences, July, 1851.

fluid drank, but that the sacharine contents in a given time underwent little or no variation. And equally so with respect to food; for though the symptoms are ameliorated when the diet is made to consist of animal matters, "there is no real improvement, and the deprivation of sacharine and amylaceous matters is not counterbalanced by the diminished thirst and less frequent calls for micturition."

We have been most struck, however, with what is said upon the effects of remedies in the treatment of diabetes, and it is this part of the article that we would reproduce here. Many remedies were tried, and their effects carefully noted for a week at a time, every precaution being taken to preserve the patient as much as possible in the same prdicament. The remedies were given in the dose indicated below, and repeated thrice daily, and the result is shown in the corresponding figures, which indicate the amount of sugar passed in the stools as well as in the urine during the week in which each remedy was tried.

| | | Grains | | Grains. |
|---|---|---|---|---|
| Strychnine | 1–6 gr. | 3369 | Without medicine . . | 14520 |
| ” | 1–7 gr. | 3565 | Creosote and naphtha . | 15028 |
| ” | 1–10 gr. | 6250 | Cod-liver oil, 6 ℥ per week | 15058 |
| ” | 1–15 gr. | 6425 | ” 10 ℥ ” | 16108 |
| ” | 1–20 gr. | 6360 | Ergot . . . . . . . | 17150 |
| Mur. tinc. ferri | 10 drops | 6900 | Cod-liver oil 20 ℥ per week | 20160 |
| ” | 20 ” | 8264 | Whiskey . . . . . . | 20504 |
| Aqua ammonia | 5 ” | 12550 | Calomel and opium . | 24230 |
| Iod. potass. | 3 gr. | 14270 | Ergot, strych., and iron | 24340 |

*Strychnine.*—The amount passed without medicine is obtained from the average of eleven analyses. We see, therefore, that the influence of strychnine exerts by far the greatest control over the quantity of sugar passed in the urine and fæces. The patient was kept under its influence for various periods, amounting in all to four months. It is here shown that, under doses of one-twentieth of a grain, the amount is diminished to less than one-half, and under one-sixth of a grain to less than one-fourth. For three successive days he was kept upon a meat diet, and one-sixth of a grain of strychnine administered three times daily. The quantity of sugar, on the third day, was diminished to 132 grains. This was the 30th of October, and was the smallest quantity we ever found in this patient's urine.

*Tinc. Ferri. Mur.*—This remedy, in doses of ten drops, diminished the sugar one-half; but on increasing the dose to twenty drops, a notable increase manifested itself, though still showing the beneficial effects of the medicine.

*Aqua Ammonia.*—The diminution here amounted to one-seventh. Larger doses were tried, but they produced so much uneasiness that they had to be discontinued.

*Iodide of Potass.*—The effect of this remedy over the excretion of sugar was little or none. It produced pain in the bowels and diarrhœa.

*Creosote and Naphtha.*—These also produced great inconvenience, and their effect was to increase slightly the quantity of sugar.

*Cod-liver Oil.*—It whatever doses this medicine was administered, its effect was to increase the amount of sugar. When six ounces per week were taken, the difference was slight; but when increased to twenty, one-third more sugar was passed. One fact, however, is worthy of notice. The patient, under this remedy always gained weight, and, with the exception of the period when ergot was administered, only at that time. In forty-four days, on four pounds of oil, he gained nineteen pounds.

*Ergot.*—The patient under the influence of this remedy, gained in one week nine and a half pounds, but the amount of sugar increased one-sixth.

*Whiskey.*—This increased greatly the quantity of urine, as might be supposed, and also the sugar, which amounted to one-third more than when he was taking no medicine.

*Calomel and Opium.*—This was continued for two weeks, till the patient was brought decidedly under the influence of the mercury. The calls to urinate became more frequent, and the amount of sugar became nearly doubled.

*Ergot, Strychnine, and Iod. Ferri.*—Under this combination the excretion of sugar was about the same as the preceding. He complained greatly of the mixture, and the largest quantity of urine was passed during its administration.

